# Sequential Extraction of Hydroxytyrosol, Mannitol and Triterpenic Acids Using a Green Optimized Procedure Based on Ultrasound

**DOI:** 10.3390/antiox10111781

**Published:** 2021-11-07

**Authors:** Irene Gómez-Cruz, María del Mar Contreras, Inmaculada Romero, Eulogio Castro

**Affiliations:** 1 Department of Chemical, Environmental and Materials Engineering, Universidad de Jaén, Campus Las Lagunillas, S/N, 23071 Jaén, Spain; igcruz@ujaen.es (I.G.-C.); iromero@ujaen.es (I.R.); ecastro@ujaen.es (E.C.); 2 Centre for Advanced Studies in Earth Sciences, Energy and Environment (CEACTEMA), Universidad de Jaén, Campus Las Lagunillas, S/N, 23071 Jaén, Spain

**Keywords:** exhausted olive pomace, green extraction, hydroxytyrosol, mannitol, maslinic acid, ultrasound-assisted water extraction

## Abstract

Olive-derived biomasses contain bioactive compounds with health promoting effects as well as antioxidant and sweet-tasting properties. However, their sequential extraction has not been attained. In the present study, firstly antioxidants and mannitol were extracted from exhausted olive pomace (EOP) by an eco-friendly method, ultrasound-assisted water extraction (UAWE). The amplitude (20–80%), extraction time (2–18 min) and solid loading (2–15%, *w*/*v*) were evaluated according to a Box–Behnken experimental design. Using the response surface methodology, the optimal conditions for extraction were obtained: 80% amplitude, 11.5% solid loading and 16 min. It enabled the multi-response optimization of the total phenolic content (TPC) (40.04 mg/g EOP), hydroxytyrosol content (6.42 mg/g EOP), mannitol content (50.92 mg/g EOP) and antioxidant activity (ferric reducing power or FRAP, 50.95 mg/g EOP; ABTS, 100.64 mg/g EOP). Moreover, the phenolic profile of the extracts was determined by liquid chromatography-UV and mass spectrometry, identifying hydroxytyrosol as the main phenolic compound and other minor derivatives could be characterized. Scanning electron microscopy was used to analyze the morphological changes produced in the cellular structure of EOP after UAWE. In addition, the chemical composition of the extracted EOP solid was characterized for further valorization. Then, a second extraction step was performed in order to extract bioactive triterpenes from the latter solid. The triterpenes content in the extract was determined and the effect of the previous UAWE step on the triterpenes extraction was evaluated. In this case, the use of ultrasound enhanced the extraction of maslinic acid and oleanolic acid from pelletized EOP with no milling requirement. Overall, UAWE can be applied to obtain antioxidant compounds and mannitol as first extraction step from pelletized EOP while supporting the subsequent recovery of triterpenic acids.

## 1. Introduction

Hydroxytyrosol is one of the natural antioxidants present in olive-derived biomasses and olive oil [1]. In particular, it is found in different by-products/wastes obtained during olive oil and pomace olive oil production such as olive leaves, olive pomace, exhausted olive pomace (or extracted olive pomace, defatted olive pomace) (EOP) and olive mill wastewater [2]. Different preclinical studies have attributed anti-inflammatory, antiproliferative, proapoptotic, antimicrobial and neuroprotective properties to hydroxytyrosol [3,4,5]. The healthy benefits of hydroxytyrosol have also been shown in clinical trials [6]. It protects against lipid oxidation and prevents cardiovascular disease [6] and in combination with vitamin E improves steatosis and hypertriglyceridemia in children [7]. In fact, a health claim has been published by the European Food Safety Authority (EFSA) on the benefits of a minimum daily intake of 5 mg hydroxytyrosol and its derivatives through olive oil consumption [6,8].

Furthermore, hydroxytyrosol has a lipophilic and a hydrophilic feature that makes it soluble in water and fats [1]. In this way, the EFSA has included hydroxytyrosol as a new safe antioxidant food ingredient to be added into fish and vegetable oils and spreadable fats [9].

In olive-derived biomasses, other bioactive compounds can be found, e.g., the sugar alcohol mannitol and the triterpenes maslinic acid and oleanolic acid [10,11,12]. The former compound has food preservative properties (increasing food shelf life by reducing sugar crystallization) and it is a low-calorie sweetener [11,13]. It is also currently used as a drug to treat acute stroke and as a protective and therapeutic agent during neurological or renal failure [14]. Mannitol can be co-extracted with phenolic compounds using alcoholic-water solutions [10]. In the case of the triterpenic acids, they also have important health and disease prevention properties [15], such as anti-inflammatory, antihyperlipidemic, antitumoral and antimicrobial activities [16,17,18,19]. Triterpenic acids generally require pure or closely pure alcohols for solubilization, as shown some previous studies on olive leaves [20] and olive pomace [21].

Currently, novel extraction methods such as ultrasound-assisted extraction (UAE) are applied to obtain bioactive compounds from agri-food biomasses. Ultrasound is one of the key technologies to achieve the goal of sustainable “green” extraction and chemistry. UAE is a non-thermal technology that reduces the extraction time and the solvent-to-solid ratio compared, for example, with Soxhlet extraction [22,23,24], as it is an environmentally and economically viable alternative to conventional extraction techniques [24,25]. UAE can produce cavitation, vibration and mixing of the media. These effects cause the cell wall of the materials to rupture and allow the extraction of the compounds of interest [24,26]. Industrial-scale systems are currently available, including bath systems from TierraTech and probe systems (batch and continuous) from Hielscher-Ultrasound Technology [27]. Thus, the evaluation of the operational parameters affecting the extraction of the latter bioactive compounds at lab scale can be useful to move towards large scale and valorize EOP, the industrial final waste obtained in the olive sector in some countries.

In a previous work on EOP, water was used as an efficient and environmentally friendly solvent to recover phenolic compounds from EOP, but both high temperature and long extraction time were required, i.e., 85 °C and 90 min, respectively [28]. This work means an advance in the study of this little explored waste for the recovery of bioactive compounds, including phenolic compounds and mannitol. To the best of our knowledge, this is the first time that ultrasound assisted water extraction (UAWE) of EOP has been optimized and the main operational parameters evaluated. In addition, a second ethanolic extraction step was used to recover triterpenic acids from this feedstock. This sequential extraction extends the opportunities to exploit EOP in a greener manner.

## 2. Materials and Methods

### 2.1. Chemicals, Reagents and Standards

Folin–Ciocalteu’s phenol reagent, 2,2′-azinobis(3-ethylbenzothiazoline-6-sulfonate) (ABTS™), 2,4,6,-tri(2pyridyl)-1,3,5,-triazine (TPTZ), 6-hydroxy-2,5,7,8-tetramethylchroman-2-carboxylic acid (Trolox), orthophosphoric acid and the standard of gallic acid were provided by Sigma-Aldrich (St. Louis, MO, USA). Methanol and acetonitrile of HPLC grade was obtained from Honeywell (Morristown, NJ, USA) and PanReac AppliChem (Barcelona, Spain), respectively. The following standards were obtained from Extrasynthese (Genay, France): hydroxytyrosol, maslinic acid and oleanolic acid. Ultrapure water was obtained using a Milli-Q system (Millipore, Bedford, MA, USA).

### 2.2. Raw Material

Industrial EOP was obtained as pellets (average length, 14.5 mm; average diameter, 4.6 mm) from “Spuny SA” (Linares, Jaén, Spain). One part of the sample was milled to a particle size of around 1 mm in an Ultra Centrifugal Mill ZM 200 (Retsch, Haan, Germany) and the other part remained pelletized. Both samples were stored in a dry place until use.

### 2.3. Methodology

**Figure 1:**
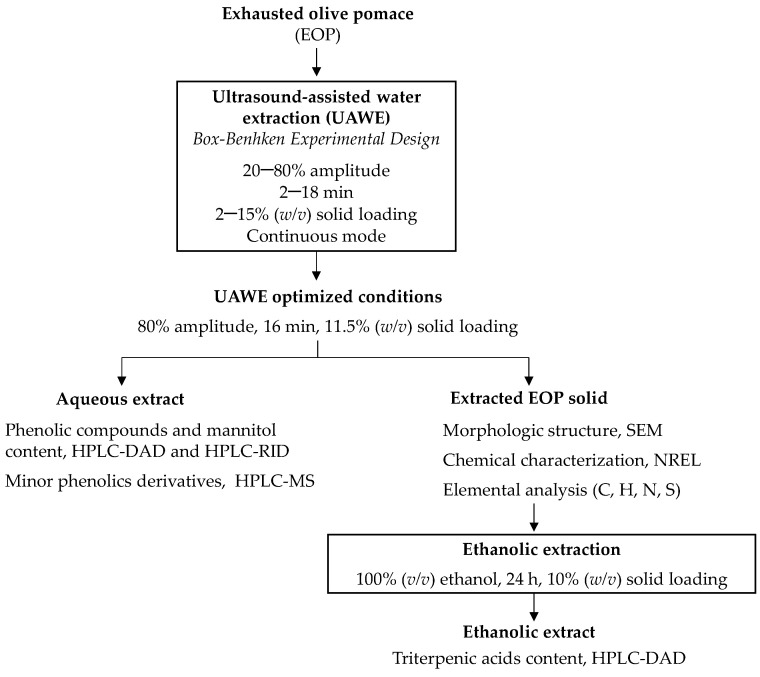
Scheme of the experimental procedure and the characterization techniques.

Figure 1 shows the experimental procedure followed in this work in order to extract phenolic compounds and mannitol from EOP by UAWE and to optimise the experimental conditions. After that, triterpenic acids were recovered in a second ethanolic extraction and the resulting fractions were characterized.

Firstly, the effect of milling was tested, while using a Box–Behnken design (BBD), UAWE operational parameters (extraction time, solid loading, and amplitude) were evaluated to obtain phenolic compounds and mannitol. Moreover, the extracts obtained under optimized conditions were further characterized by high-performance liquid chromatography (HPLC)-ion trap mass spectrometry (IT-MS) to profile minor hydroxytyrosol derivatives. The structural changes of the extracted solid obtained under UAWE optimized conditions were studied by scanning electron microscopy (SEM) and it was also chemically characterized. Then, that solid was subjected to a second extraction step using ethanol to obtain maslinic acid and oleanolic acid and the effect of the previous step was evaluated.

### 2.4. UAWE of EOP

UAWE was performed using an ultrasonic probe (Branson 550, Ultrasonics Corporation; Danbury, CT, USA) with a power of 550 W and a frequency of 50–60 KHz. A sonotrode of 30 mm of diameter was used. The sonotrode was immersed 1 cm deep into the samples (250 mL in vessels). The sonication was performed in continuous mode, i.e., the waves propagated continuously through the sample during the whole extraction time, without any interruption. The experiments were initiated at room temperature and the increment of temperature due to the sonication effects was measured at the end of each assay.

After extraction, the slurry was filtered under vacuum, obtaining two fractions: a liquid fraction (aqueous extract) and a solid fraction (extracted EOP solid). An aliquot of the aqueous extracts was filtered with a syringe filter (nylon, pore size 0.45 μm) (SinerLab Group, Madrid, Spain) and stored at −20 °C until analysis. The solid was oven-dried at 40 °C and then stored.

Firstly, some preliminary experiments were carried out with pelletized and milled EOP, using a solid loading of 8.5% (*w*/*v*) to test two amplitudes (20% and 80%) and extraction times (2 min and 13 min). All extraction assays were conducted in duplicate. Then, an experimental design was applied (Section 2.5).

### 2.5. Box-Behnken Experimental Design for UAWE of EOP

The optimization of UAWE of bioactive compounds from the pelletized EOP was performed using a response surface methodology (RSM). As operational variables, ultrasound amplitude (20–80%), extraction time (2–18 min) and solid loading (2–15%, *w*/*v*) were studied. The BBD consisted of 17 experiments, including five central points (50% amplitude, 10 min and 8.5% of solid loading). These experiments were performed in random order.

The influence of each independent variable was determined according to the following equation:

(1) $$y_{j}=\beta_{0}+\overset{3}{\underset{i=1}{\sum }}\beta_{i}x_{i}\;+\;\sum \overset{3}{\underset{i<j=1}{\sum }}\beta_{ij}x_{i}x_{j}+\overset{3}{\underset{i=1}{\sum }}\beta_{ii}x_{i}^{2}$$

where *y* is the response variable, *x_i_* and *x_j_* are the operational variables in coded values ranging from −1 to 1, *β_0_*, *β_i_*, *β_ij_* and *β_ii_* are the regression coefficients calculated from the experimental results by the least-squares method. Analysis of variance (ANOVA) was used to establish the significance of the results. To determine the model goodness, the following parameters were evaluated: the coefficient of determination (R^2^), adjusted determination coefficient (R^2^ adj) and coefficient of variance (CV, percent). The significance of all terms in the polynomial equation was considered statistically different when *p* < 0.05. After applying a multiple response optimization, the optimal conditions were reproduced (five replicates) to compare the experimental with the predicted data and assess the validity of the model. Additionally, these conditions were applied to obtain antioxidant compounds and mannitol from milled EOP for comparison.

After each extraction, the samples were filtered under vacuum, treated and stored as previously described.

### 2.6. Extraction Yield

It was determined gravimetrically. In brief, an aliquot of the extracts (2 mL) was poured into a 10 mL glass tube. Then, it was oven-dried at 105 °C until constant weight. All samples were measured in duplicate. The data were expressed as percent (g of extract/100 g of EOP, dry weight).

### 2.7. Analytical Determinations of the UAWE Extracts

#### 2.7.1. Total Phenolic Content and Antioxidant Capacity

TPC was estimated using the Folin–Ciocalteu assay according to Gómez-Cruz et al. [28]. The absorbance of each sample was measured at 760 nm in a Bio-Rad iMark^TM^ microplate absorbance reader (Hercules, CA, USA). Gallic acid was used as standard (concentration range 0–0.295 g/L) and the results expressed as grams of gallic acid equivalents (GAE)/L in the aqueous extract and milligrams of GAE/g of EOP (dry weight).

In addition, the antioxidant activity of the extracts obtained from EOP was determined by ferric reducing power assay (FRAP) and ABTS assay according to Gómez-Cruz et al. [28] at 734 nm and 593 nm, respectively, using the aforementioned device. The first method is based on the reduction of Fe(TPTZ)_2_^3+^ to Fe(TPTZ)_2_^2+^ by the donation of electrons from the antioxidant compounds and the second method is based on the neutralization of the ABTS radical by the antioxidant compounds. For both assays, the results were expressed as mg Trolox equivalent (TE)/g of EOP (dry weight) using Trolox as standard (concentration range, 0–0.279 g/L for FRAP; 0–0.629 g/L for ABTS). TPC and antioxidant assays were carried out in triplicate.

#### 2.7.2. Characterization of Phenolic Compounds and Mannitol by HPLC Analyses

For the determination of phenolic compounds, a Shimadzu Prominence HPLC equipment was applied according to Gómez-Cruz et al. [28]. It was equipped with an SPD-M20A diode array detection (DAD). The analysis was performed using a BDS HYPERSIL C18 column (290 mm × 4.6 mm, 5 μm particle size) (Thermo Fisher Scientific Inc., Waltham, MA, USA). Retention time and UV absorption spectra allowed the identification of hydroxytyrosol by comparison with its commercial standard. This compound was quantified at 280 nm (*y* = 19,113*x* − 15,977; R^2^, 1.0000) and the results were expressed in g/L in the aqueous extract and mg/g of EOP (dry weight).

In addition, HPLC-IT-MS and -MS^2^ analyses were performed in an Agilent 1100 HPLC connected on-line to an Esquire 6000 IT (Bruker, Bremen, Germany) via an electrospray interface, according to Medfai et al. [29]. A Kinetex core–shell C18 column (2.1 mm × 50 mm, 2.7 µm) (Phenomenex, Barcelona, Spain) was applied. MS and MS/MS spectra were recorded over the mass-to-charge (*m*/*z*) range of 100–1200 in the negative ionization mode. Auto MS/MS analyses were performed at 0.6 V. DataAnalysis (version 4.0) from Bruker was used to process the data.

For mannitol analysis, samples were previously conditioned with ion-exchange resins (Microionex MB200, Rohm Haas, Denmark) to remove impurities and then, filtered through 0.45 µm nylon membranes. It was then analyzed by HPLC equipped with refractive index detection (RID). Carbohydrate column (CARBOSep CHO-782 Pb, Transgenomic, Inc., Omaha, NE, USA) and ultrapure water as mobile phase were employed. The flow rate and the column temperature were set at 0.6 mL/min and 70 °C, respectively. The results were expressed as g/L in the aqueous extract and mg/g of EOP (dry weight) using the following curve (*y* = 8.82 × 10^5^*x* + 6.63 × 10^4^; R^2^, 0.9997).

### 2.8. Characterization of the Extracted EOP Solids after UAWE

#### 2.8.1. Chemical Characterization

The methodology of the National Renewable Energy Laboratory (NREL) was used to chemically characterize the extracted EOP solids. First, the content in extractives was determined by Soxhlet extraction with water and ethanol according to NREL/TP-510-42619 [30]. Then, cellulose, hemicellulose and lignin contents were also determined according to the NREL methodology [31] and ash content according to NREL/TP-510-42622 [32]. Moreover, the elemental composition (C, H, N and S) was analyzed using a TruSpec Micro device (Leco, St. Joseph, MI, USA).

#### 2.8.2. Scanning Electron Microscopy

SEM (MERLIN from Carl Zeiss) (Oberkochen, Germany) was used to evaluate the morphological changes of the biomass before and after UAWE for both pelletized and milled EOP. Dry samples were fixed with double-sided adhesive tape mounted on SEM holders and metalized with gold. The samples were photographed at high vacuum, 5 kV and 100× and 1000× magnification, i.e., 500 μm and 50 μm scales, respectively.

### 2.9. Extraction of Triterpenic Acids from the Extracted EOP Solids after UAWE

The extraction of triterpenic acids was carried out according to Romero et al. [33], with some modifications. The extractive agent was absolute ethanol and the extraction was performed at 10% (*w*/*w*) solid loading and room temperature (24 h, 150 rpm) in a rotary shaker (INFORS HT Ecotron, Surrey, UK). Each sample was centrifuged (MicroCen 16, Herolab, Germany) and filtered with a syringe filter (nylon, pore size 0.22 μm) (SinerLab Group, Madrid, Spain).

Extracted EOP solids (solid remaining of EOP) obtained after UAWE in the previous BBD experimental assays and under optimized conditions were subjected to a second extraction for recovering triterpenes acids. All extraction assays were conducted in duplicate and the extraction yield was determined as for the aqueous extracts obtained by UAWE. Triterpenic acids were quantified using external standards: maslinic acid (*y* = 8118.3*x* + 123,127; R^2^, 0.9968) and oleanolic acid (*y* = 10,717*x* + 48,413; R^2^, 0.9992). This determination was performed using the aforementioned HPLC-DAD conditions at 210 nm, according to Romero et al., (2017). The results were expressed as g/L in the ethanolic extracts and mg/g of EOP (dry weight).

### 2.10. Statistical Analysis

The statistical analysis of the experimental design was performed using Design-Expert^®^ v8.0.7.1 software (Stat-Ease, Inc., Minneapolis, MN, USA), which also served to evaluate how the operational parameters affected the increment of temperature promoted by UAWE. In addition, the indirect effects of the UAWE operational variables on triterpene extraction were also studied with the latter software. An ANOVA analysis was also carried out using the post hoc test Fishers Least Significant Difference LSD using Statgraphics Centurion XVII (StatPoint Technologies, Inc., Warrenton, VA, USA). A correlation test and *t*-test were performed using Microsoft Office Excel 2007 (Redmond, WA, USA).

## 3. Results

### 3.1. Optimization of UAWE

#### 3.1.1. Effect of Milling on the Recovery of Antioxidants

Ultrasound is an efficient technique for the extraction of phenolic compounds from olive-derived biomasses in a short time [11,34,35], although in these works, alcoholic solutions were applied as extractive agents. In a previous work on EOP, it was evidenced that water can be applied to extract hydroxytyrosol from EOP but a relatively high temperature (85 °C) and a long extraction time (90 min) were required [28]. Thus, in the present work, water was used as the extractive agent, which is an environmentally friendly alternative to organic solvents. In addition, ultrasound was applied to assist the extraction without direct heat requirement and to shorten the extraction time.

First, preliminary tests were performed with pelletized EOP and milled EOP (~1 mm), in order to evaluate whether the milling step can be avoided when using UAWE. The solid loading was fixed at 8.6% (*w*/*v*) and the extraction amplitude and time were varied (Table 1). Pelletized and milled EOP were ultrasound-assisted extracted at different conditions: (i) 20% amplitude and 2 min (P1, M1) and (ii) 80% amplitude and 13 min of extraction time (P2, M2). The results showed that at low amplitudes and short extraction times, higher yield and solubilization of phenolic compounds, including hydroxytyrosol, and mannitol are achieved if the EOP is milled (M1), with an increase higher than 100%. However, at a higher amplitude and longer extraction time, the extraction results of pelletized EOP (P2) were slightly lower or similar to that of milled EOP (M2). In this sense, one mechanism related to the employment of ultrasound is the fragmentation generated by the collisions between particles and ultrasonic waves. It can provoke a reduction of the particle size and so facilitating mass transfer [36]. Since amplitude is the maximum height of a sound wave and it is related to the ultrasound intensity [37], this can explain the results obtained. Therefore, EOP was kept pelletized, as found at industrial scale, to optimize the UAWE without the application of the milling step.

#### 3.1.2. Fitting the Models

In the present study, a BBD of experiments was used to optimize and study the influence of the amplitude, extraction time and solid loading on the extraction of bioactive compounds from pelletized EOP using UAWE. The extraction yield, phenolic concentration, TPC, mannitol concentration and content, hydroxytyrosol concentration and content, and antioxidant activity of the extracts were chosen as response variables. Table 2 shows the results obtained for the experimental design.

Then, the results were analyzed by multiple regression fitting to obtain the equation describing the relationship between each variable response and the operational variables, according to Equation (1) (Table 3). The regression coefficients determined by ANOVA for each model, and the statistical parameters F-values, R^2^, R^2^ adj, CV and lack of fit (*p*-value) are detailed in Table 3. The F-value for the response variables (13.83–215.76) and the *p*-value < 0.05 indicated that the models were statistically significant. The R^2^ for all responses was good (0.874–0.997), indicating an adequate fitting of the models. The R^2^ adj values were also adequate, suggesting a high degree of correlation between the predicted and experimental values. The CV was generally lower than 10% confirming an adequate accuracy of the models. Furthermore, the *p*-value for the lack of fit was higher than 0.1, which means that the dispersion of the experimental data was a measure of pure error independent of the model.

#### 3.1.3. Response Surface Analysis

##### Influence of the Extraction Conditions on the Solubilization of Bioactive Compounds

**Figure 2:**
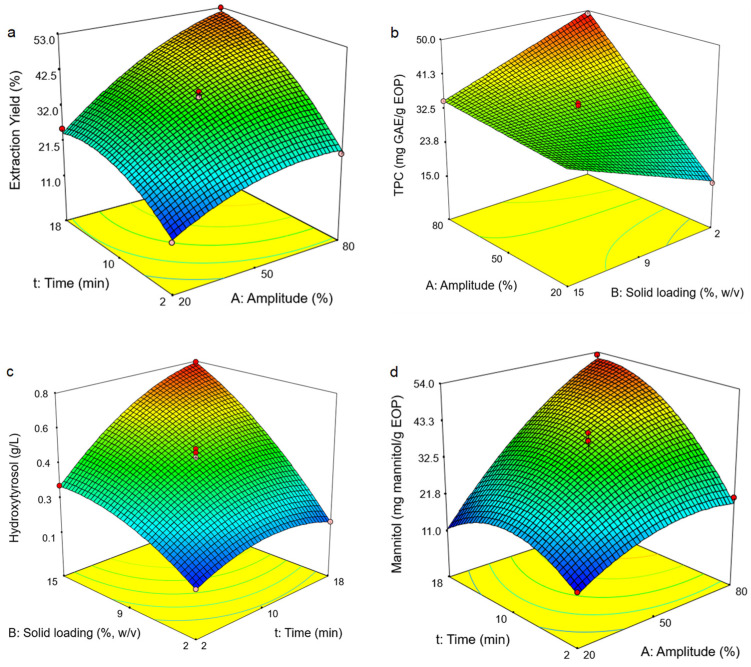
Response surfaces obtained by the Box–Behnken design for exhausted olive pomace: (**a**) extraction yield, (**b**) total phenolic content (TPC), (**c**) hydroxytyrosol concentration and (**d**) mannitol content. The solid loading was fixed at 8.5% (*w*/*v*) in plot (**a**,**d**), the extraction time at 10 min in plot (**b**) and the amplitude at 50% in plot (**c**).

According to the mathematical model, the extraction yield (Table 3, Equation (2)) depends mainly on the extraction amplitude and extraction time; the linear terms of both parameters being positive and of similar influence, but their quadratic terms resulted in nonlinear curves. Time and amplitude has a synergistic effect and therefore the combination of the two parameters improved the extraction yield, as Sharayei et al. [23] suggested for the extraction of bioactive compounds from pomegranate peel. As an example, Figure 2a shows the positive influence of both amplitude and time on the extraction yield at 8.5% (*w*/*v*) solids and the presence of curvatures, reaching a plateau. The solid loading showed a negative influence on this response, but less significant.

For the phenolic concentration and TPC, the Equations (3) and (4) (Table 3) show that the three linear terms of the independent variables had a positive influence on these responses. Nonetheless, the solid loading has a greater influence for the former and the TPC showed a main dependency on the linear terms of the amplitude and extraction time. The amplitude and extraction time also showed a positive interaction as before and curvatures in both cases. Moreover, a negative interaction between the amplitude and the solid loading was observed for this response as shown Figure 2b.

The response of hydroxytyrosol concentration is similar to that of phenol concentration. All the three independent variables (linear terms) show a positive influence, with the influence of solid loading being the greatest (Equation (5)). This trend was similar (Equation (7)) for the mannitol concentration (Table 3) and a positive and synergistic effect between the factors on both response variables was observed in both cases (as an example, see Figure 2c).

Alternatively, the content of hydroxytyrosol and mannitol, expressed as mg/g EOP, showed similar behavior as the extraction yield and TPC responses (Equations (6) and (8), respectively) (Table 3). The linear terms of the amplitude and extraction time have a positive influence for both variables, reaching a plateau, but the mannitol content mainly depends on the amplitude. Alternatively, the linear term of the solid loading has a negative influence. As an example, Figure 2d shows the effect of the amplitude and extraction time on both responses, keeping the solid loading at a medium level (8.5%, *w*/*v*), as before. Their positive interaction was highly remarkable in the case of the latter response variable.

In previous studies, the ultrasonic radiation amplitude and extraction time were evidenced to be influential factors for obtaining sugars and mannitol from olive matrices (leaves and fruits) [38]. Both are also key factors to be optimized for promoting the extraction of bioactive compounds from agri-food residues [36,39,40,41]. Although increasing these parameters can favor the diffusion of target compounds, longer extraction times can promote the decomposition of these bioactive compounds, for example, phenolic compounds [42]. This can explain that generally a maximum or a plateau was achieved at values closer to 18 min. Additionally, related to the amplitude percentage (or rated power) is that the resonant bubble size is proportional to the power of the ultrasonic wave. On the one hand, as the bubble size increases, its impact on implosion is also enhanced, and on the other hand, the hydrodynamic force can also be increased. The former effect can enhance the diffusivity and extraction efficiency due to fragmentation, pore formation, and mixing, while the latter is also related to the disruption of the plant matrix [43]. Alternatively, a high amplitude may result in low transmission of the ultrasound waves due to two potential effects: solvent agitation instead of cavitation and saturation because the cavitation bubbles are assembled around the probe [43]. Under the conditions tested in this work, a plateau is reached in most of the response variables due to the intensification effect of the amplitude, which could be related to it. Other authors have also observed that the degradation of the bioactive compounds occurred when increasing the amplitude percentage [44].

An increment of temperature was also observed during the UAWE since the assays were carried out without temperature control. This fact agreed with previous studies [41,45]. This means that, besides the effect of the aforementioned parameters on the recovery of bioactive compounds, a joined effect of the temperature should not be ruled out. Temperature could participate favoring the extraction as has been evidenced in a previous work for EOP antioxidants [28], but it is incited by the ultrasound treatment [45]. The formation of small bubbles due to ultrasound is subjected to fast adiabatic compression and expansion, which generates a fast local increase of temperature and pressure. It can be related to the breakage of the plant cell wall, and thereby the extraction efficiency [46,47]. In this sense, the increment of temperature can be modeled taking into account the operational parameters amplitude, extraction time and solid loading, being mainly influenced by the amplitude percentage and extraction time; both the linear and quadratic terms showed *p*-values lower than 0.05 (Table S1). This is also shown by Figure S1 and thus the effect of the amplitude percentage and extraction time cannot be separated from that of the temperature.

##### Influence of the Extraction Conditions on the Antioxidant Activity

**Figure 3:**
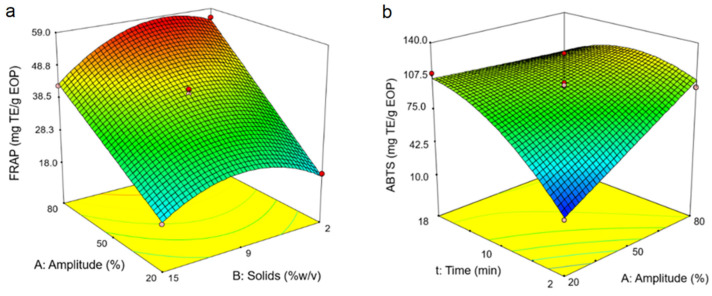
Response surfaces obtained by the Box–Behnken design for exhausted olive pomace: (**a**) ferric reducing power (FRAP) assay; (**b**) ABTS assay. The time was fixed at 10 min in plot (**a**) and the solid loading at 8.5% (*w*/*v*) in plot (**b**).

Two common assays were performed to evaluate the impact of the UAWE on the antioxidant capacity of the EOP extracts. The software generated different model equations for the FRAP and ABTS assays, i.e., Equations (9) and (10), respectively (Table 3). For both responses, the linear terms of the amplitude and extraction time have a positive influence, while solid loading has a negative influence. As examples, Figure 3a shows the response surface for amplitude and solid loading at 10 min and Figure 3b represents the amplitude and extraction time at a fixed solid loading of 8.5%.

The antioxidant capacity, determined by both methods, correlated well with the yield, TPC, hydroxytyrosol and mannitol content, due to a generally similar behavior of the response variables during UAWE of EOP; i.e., Pearson correlation values ranged from 0.934 to 0.985 for the FRAP assay and from 0.763 to 0.785 for the ABTS assay.

##### Process Optimization and Validation of the Model

A multiple response optimization was applied to maximize all the latter variables at a time and to reproduce the optimal conditions in the laboratory, which were: 80% amplitude, 16 min and 11.5% solid loading. These values are within the range of the previous values for the individual optimization. The results are shown in Table 4, highlighting that the experimental data were similar to the predicted values; the error was generally less than 10% in all cases. Not surprisingly, these values are slightly lower to those obtained by optimizing each response variable separately, but it is a good compromise to obtain an EOP extract with high values for all of them.

The extraction yield was 47.12% and the extract presented a TPC value of 40.04 mg GAE/g of EOP. These values compare favorably with those obtained from water extraction of EOP at 85 °C and 90 min with an extraction yield of 40.9% and a TPC value of 44.5 mg GAE/g EOP [28]. The hydroxytyrosol concentration was slightly higher using UAWE as compared to the latter conventional method, 0.74 g/L vs. 0.6 g/L, respectively [41]. Moreover, all these values for EOP are higher than those reported in extracts from olive pomace obtained using aqueous organic solvents and UAE and pressurized-liquid extraction [35,48] or in the liquid fraction recovered by centrifuging this by-product [49]. In the case of mannitol, previous studies reported values between 39 mg/g of EOP and 45 mg/g of EOP from an aqueous extraction at 85 °C and 90 min and 100 °C and 30 min, respectively [28,50]. The extracted mannitol value is in the range of those values reported for olive leaves using pressurized liquid extraction and Soxhlet extraction [51] and slightly lower than using centrifugation on olive pomace [49]. Overall, EOP is a natural source of these valuable compounds and UAWE can be applied as an eco-friendly extraction strategy.

Therefore, the use of ultrasound allowed shortening the extraction time and the temperature requirements, obtaining higher or similar values to those obtained using conventional methods and water as extraction agent. Nonetheless, at the end of the UAWE assay a temperature of 75 °C was reached due to the ultrasound application, which corresponds to an increment of temperature around 55 °C; as we commented before, heating is produced by the ultrasound treatment.

In addition, although the design was performed with pelletized EOP, these conditions were also applied to milled EOP for comparison. Under the optimized conditions for pelletized EOP, the milling step can be saved as shown in Section 3.1.1.

### 3.2. Phenolic Profiles and Standardization

**Figure 4:**
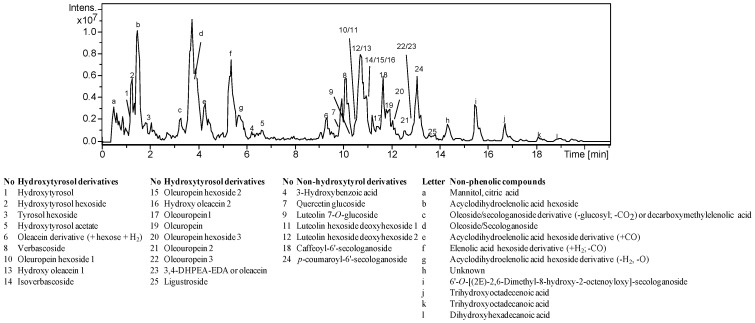
Base peak chromatogram and compounds characterized in the aqueous extracts obtained from pelletized exhausted olive pomace after ultrasound-assisted water extraction at 80% amplitude, 16 min and 11.5% solid loading (*w*/*v*).

The aqueous extracts obtained from pelletized and milled EOP under the optimal UAWE conditions were analyzed by RP-HPLC-DAD and HPLC-MS and the extracts showed similar phenolic profiles. The main compound identified at 280 nm was hydroxytyrosol, which is one of the main compounds found in olive pomace (Figure S2) [35,48,52]. Moreover, when the extracts were analyzed by HPLC-MS, other minor compounds were characterized according to previous studies [11,53]. Both extracts presented 25 phenolic compounds, among other compounds like mannitol, citric acid, and monoterpene and fatty acids derivatives (as an example, Figure 4). The phenolic compounds were mainly derivatives of hydroxytyrosol, but also phenolic acids and flavonoids were detected. The hydroxytyrosol cluster includes oleacein, verbascoside and oleuropein, which are also relevant bioactive compounds found in olive oil and olive leaves, respectively [54,55,56].

### 3.3. Characterization of the Extracted EOP Solids

#### 3.3.1. Chemical Characterization and Elemental Analysis

Table 5 shows the chemical and elemental composition of pelletized EOP and milled EOP for comparison. On the one hand, the UAWE step for both pelletized and milled EOP led to the removal of a part of the extractives (non-structural components) obtaining extracted solids with lower amounts of this fraction. On the other hand, as a consequence of the solubilization of the aqueous extractives and ashes, there is an increase in the percentages of the ethanolic extractives, carbohydrates and lignin of the extracted solids for both pelletized and milled EOP; in all cases this increase was higher than 38%. Among the hemicellulosic sugars, the largest increase was observed for its main sugar, xylose.

The study by Manzanares et al. [50] has shown an extracted EOP solid with a lower amount of extractives (14.9%), but applying aqueous extraction at more severe conditions (100 °C, 30 min). In other work, using acetone–water (40:60, *v/v*) as the solvent to extract phenolic compounds, the release of extractives depended on the conditions applied, including if it was bath- or probe-type UAE [41]. The percentages of cellulose and hemicellulose in the extracted solids are closer to that found in these studies. In all cases, these lignocellulosic-enriched solids can be useful for further valorization to obtain sugars and derivatives and lignin or energy.

In terms of the elemental composition of the solids, compared to the raw EOP [57], UAWE meant an increase in all components (nitrogen, carbon and hydrogen), with the most considerable increase in sulfur, which had not been previously detected. This relative increase in sulfur could be related to the increase of the proteins percentage (considering the nitrogen content) in the extracted EOP solids, as it is a mineral that is generally bound to proteins [58].

#### 3.3.2. SEM Analysis of Raw EOP and Extracted EOP Solids

**Figure 5:**
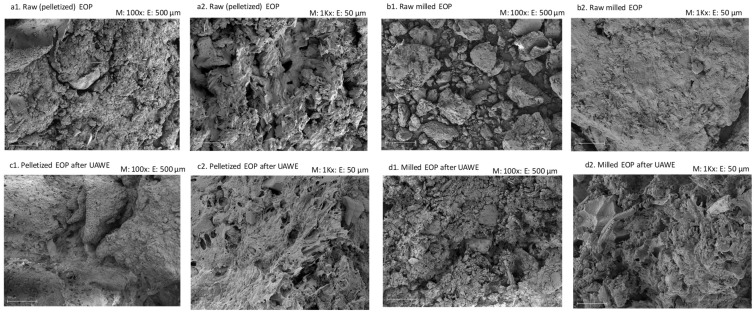
Scanning electron microscopy images at different magnifications of raw (pelletized) (**a1**,**a2**) and milled (**b1**,**b2**) exhausted olive pomace (EOP) and of their respective extracted EOP solids (**c1**,**c2**,**d1**,**d2**, respectively) obtained after ultrasound-assisted water extraction (UAWE) at 80% amplitude, 16 min and 11.5% solid loading (*w*/*v*).

The cellular structure of the raw (pelletized) and milled EOP before and after UAWE was investigated by SEM. The panoramic images (at 100× magnification, 500 μm scale) of the raw pelletized and milled EOP showed a predominance of plant material (Figure 5(a1,b1), respectively). Not surprisingly, the milled EOP presented a more disaggregated structure due to the milling conditioning step.

Figure 5(c1,d1) are also panoramic images of the pelletized and milled EOP, respectively, after being subjected to UAWE under optimized conditions (80% amplitude, 16 min and 11.5% (*w*/*v*) solid loading). In Figure 5(c1), the plant material surface appears more eroded than the raw pelletized EOP surface (Figure 5(a1)) and had a frayed appearance due to the ultrasonic treatment. This is specially highlighted in Figure 5(c2) when compared to Figure 5(a2), with a magnification factor of 1000× (scale 50 μm). Again, due to the grinding step, the milled EOP after UAWE appeared more fractionated (Figure 5(d1,d2)). Despite these differences, the extraction results (Table 4) were similar in the EOP, with and without milling, and the damage provoked by the ultrasound in the pelletized EOP tissue was enough to recover phenolic compounds and mannitol.

Several authors have shown that an ultrasonic treatment causes cell damage, resulting in surface peeling, erosion and small threads or hollow openings can appear [59,60,61,62]. For example, Karki et al. [63] observed that the particulate surface of defatted soy flakes acquired a sponge-like texture after the ultrasonic treatment and the severity of disintegration improved when the amplitude and extraction time increased. These events increase the accessibility of the solvent to the plant material and thereby the rate of mass transfer of bioactive compounds [61,62], explaining our previous results.

### 3.4. Triterpenic Acids Content in Ethanolic Extracts

Triterpenic acids are another group of phytochemicals with bioactive properties [64], which have been detected in olive pomace [33] and EOP [10]. Since these compounds were not detected in the aqueous extracts obtained in this work, an extraction step with ethanol was performed and their extraction evaluated. Note that the UAWE step increased the percentage of the ethanolic extractives in the extracted EOP solids, as commented before, and these compounds are generally extracted with at least 90–100% organic solvents such as ethanol and methanol [20,33,65,66].

Table 6 shows the extraction yield and the concentration of maslinic acid and oleanolic acid for the extracted EOP solids obtained after applying the 17 experiments of the Box–Benkhen design and the ethanolic extraction described in Section 2.8. The extraction yield values ranged from 5.49% to 9.18%, the maslinic acid concentration from 0.62 g/L (or 6.22 mg/g of extracted EOP solid) to 0.90 g/L (or 9.00 mg/g of extracted EOP solid), and the oleanolic acid concentration from 0.23 g/L (or 2.30 mg/g of extracted EOP solid) to 0.33 g/L (or 3.29 mg/g of extracted EOP solid).

The Design Expert software was applied to evaluate how the UAWE operational parameters indirectly affected the extraction yield and the concentration of triterpenes from the extracted EOP solids. Table S2 shows the statistical parameters F-ratio and *p*-value for each of them. In the case of the extraction yield, the amplitude was the only significant factor. However, for the concentration of triterpenes, the extraction time was the main factor affecting the extraction with *p*-values around 0.05; the linear term affected positively and the quadratic term negatively. This indicated that intermediate values for the extraction time are recommended in the UAWE step as for the other bioactive compounds.

Moreover, Table 7 shows the comparison of the extraction yield and triterpenic concentration between the raw EOP and the extracted solids obtained from pelletized and milled EOP after UAWE at 80% amplitude, 16 min and 11.5% solid loading (*w*/*v*). The extraction yield did not increase after UAWE, while higher concentrations of triterpenic acids were obtained from the extracted EOP solids, both for pelletized (0.84 g/L; 8.41 ± 0.42 mg/g dry solid) and milled EOP (0.89 g/L; 8.95 ± 0.10 mg/g dry solid). Therefore, these results also suggest that the pelletized EOP could be used directly for the sequential extraction of bioactive compounds, i.e., phenolic compounds, mannitol, and triterpenic acids, using UAWE as the first extraction step.

Comparing the latter values with those obtained from other olive-derived biomasses, the maslinic acid content is similar to that obtained from olive leaves [20,65], but higher than that from olive pomace [33], suggesting that EOP is a source of this bioactive compound. However, the oleanolic acid content in EOP is similar to that of olive pomace [33], but olive leaves have much higher levels [20].

Finally, the concentration of hydroxytyrosol was also measured in the ethanolic extracts. In particular, the concentration was 0.073 g/L (or 0.73 mg/g dry solid) and 0.082 (0.82 mg/g dry solid) from the extracted solids obtained after UAWE of pelletized and milled EOP, respectively. This suggests that almost all hydroxytyrosol was extracted in the first UAWE due to its polar characteristic. This makes sense since in this work, a multiple optimization has been chosen instead of using the optimal individual conditions for the extraction of hydroxytyrosol and a part of hydroxytyrosol still remains in the solid.

## 4. Conclusions

The present study revealed that UAWE can be applied to extract phenolic compounds, mostly hydroxytyrosol, and mannitol from the industrial pelletized EOP. After applying the response surface methodology, the optimal conditions that enabled to maximize simultaneously the yield, the extraction of phenolic compounds, including hydroxytyrosol, and mannitol were: 80% amplitude, 16 min and 11.5% solid loading (*w*/*v*). The TPC, hydroxytyrosol content and mannitol content were 40.04 mg GAE/g EOP, 6.42 mg/g EOP and 50.92 mg/g EOP, respectively. Besides hydroxytyrosol, other minor but interesting derivatives were characterized, including oleacein, verbascoside and oleuropein. Moreover, UAWE of EOP favored the subsequent recovery of maslinic and oleanolic acids by ethanolic extraction. Overall, UAWE is a promising green extraction procedure to valorize EOP and recover different type of bioactive compounds.

## Figures and Tables

**Table 1:** Comparison of the application of milling and ultrasound in the extraction of exhausted olive pomace (EOP): yield (%), phenolic concentration (PC) (g gallic acid equivalents/L), total phenolic content (TPC) (mg gallic acid equivalents/g dry EOP), hydroxytyrosol concentration (HT) (g/L), hydroxytytrosol content (HTC) (mg/g dry EOP), mannitol concentration (MAN) (g/L), mannitol content (MANC) (mg/g dry EOP) and antioxidant activity (ferric reducing power (FRAP) and ABTS) (mg Trolox equivalents/g dry EOP). Data represent the mean value ± standard deviation (*n* ≥ 3).

Sample	Yield	PC	TPC	HT	HTC	MAN	MANC	FRAP	ABTS
Pelletized EOP									
P1 ^1^	12.82 ± 1.40 ^c^	0.64 ± 0.04 ^c^	7.62 ± 0.51 ^c^	0.17 ± 0.03 ^c^	2.00 ± 0.31 ^c^	1.35 ± 0.17 ^b^	15.88 ± 1.99 ^c^	9.27 ± 0.01 ^c^	51.38 ± 1.34 ^d^
P2 ^2^	47.56 ± 0.22 ^a^	2.83 ± 0.01 ^b^	33.28 ± 0.15 ^b^	0.63 ± 0.04 ^ab^	7.33 ± 0.45 ^ab^	4.48 ± 0.26 ^a^	52.65 ± 3.08 ^a^	38.08 ± 1.91 ^b^	169.94 ± 2.23 ^b^
Milled EOP									
M1 ^1^	39.99 ± 1.81 ^b^	2.84 ± 0.02 ^b^	33.44 ± 0.22 ^b^	0.56 ± 0.01 ^b^	6.64 ± 0.13 ^b^	3.85 ± 0.16 ^ab^	45.23 ± 1.91 ^ab^	38.89 ± 0.47 ^b^	101.36 ± 0.08 ^c^
M2 ^1^	46.21 ± 0.77 ^a^	3.28 ± 0.01 ^a^	38.60 ± 0.15 ^a^	0.70 ± 0.02 ^a^	8.15 ± 0.03 ^a^	4.38 ± 0.47 ^a^	51.47 ± 5.57 ^a^	44.57 ± 0.96 ^a^	188.15 ± 0.56 ^a^

In each column, different letters indicate significant differences between the mean values after applying an analysis of variance (*p* < 0.05) and multiple comparison by LSD test. ^1^ Amplitude, 20%; extraction time, 2 min. ^2^ Amplitude, 80%; extraction time, 13 min.

**Table 2:** Box–Behnken experimental design applied to the ultrasound-assisted water extraction of exhausted olive pomace (EOP) and experimental values for: yield (%), phenolic concentration (PC) (g gallic acid equivalents/L), total phenolic content (TPC) (mg gallic acid equivalents/g dry EOP), hydroxytyrosol concentration (HT) (g/L), hydroxytytrosol content (HTC) (mg/g dry EOP), mannitol concentration (MAN) (g/L), mannitol content (MANC) (mg/g dry EOP) and antioxidant activity (FRAP and ABTS) (mg Trolox equivalents/g dry EOP).

Run	A	t	B	∆T	Yield	PC	TPC	HT	HTC	MAN	MANC	FRAP	ABTS
1	50	10	8.5	30	36.85	2.73	32.14	0.46	5.46	3.27	38.47	39.57	103.31
2	50	10	8.5	33	37.09	2.97	34.95	0.51	5.99	3.48	40.94	43.61	105.69
3	50	18	2	41	38.34	0.63	31.27	0.13	6.28	0.67	33.50	38.27	119.25
4	50	18	15	43	37.45	4.94	32.91	0.78	5.21	5.82	38.80	46.11	89.46
5	50	10	8.5	30	38.45	2.92	34.30	0.49	5.73	3.29	38.71	42.25	106.28
6	80	10	15	48	37.98	5.19	34.59	0.78	5.21	6.08	40.53	42.57	112.43
7	80	2	8.5	12	22.85	1.59	18.70	0.26	3.10	1.96	23.06	20.62	101.53
8	50	2	2	6	24.54	0.31	15.74	0.08	3.86	0.46	23.00	16.69	76.57
9	80	18	8.5	56	52.73	3.94	46.32	0.62	7.29	4.52	53.18	57.03	152.76
10	50	2	15	7	16.15	1.96	13.03	0.32	2.12	2.65	17.67	18.06	65.21
11	20	18	8.5	19	25.25	1.75	20.56	0.28	3.34	2.20	25.88	28.12	115.67
12	50	10	8.5	32	36.75	2.73	32.09	0.45	5.30	2.95	34.71	43.80	103.78
13	20	2	8.5	3	11.55	0.69	8.10	0.12	1.46	1.04	12.24	8.48	20.10
14	50	10	8.5	28	28.77	2.22	26.16	0.36	4.21	2.46	28.94	34.78	135.64
15	20	10	15	11	17.80	1.96	13.08	0.34	2.28	2.77	18.47	18.47	60.97
16	80	10	2	45	54.58	0.98	49.06	0.17	8.32	0.95	47.50	55.98	124.56
17	20	10	2	12	23.46	0.30	15.24	0.07	3.36	0.42	21.00	19.98	86.92

A, amplitude (%); t, extraction time (min); B, solid loading (%, *w*/*v*). ∆T, increment of temperature as a difference between the initial temperature (room temperature) and the temperature reached at the end of the experiment.

**Table 3:** Mathematical models and coefficients using coded values for the studied responses in the Box–Behnken experimental design applied to the ultrasound-assisted water extraction of exhausted olive pomace.

Dependent Variable	Equation n^o^	Model	CV (%)	R^2^	R^2^ adj.	F-Value ^1^	Lack of Fit (*p*-Value)
Yield (%)	(2)	37.29 + 9.78∙A + 9.84∙t − 2.46∙B + 4.04∙A∙t + 1.87∙t∙B − 3.91∙A^2^ − 5.28∙t^2^ − 2.88∙B^2^	4.50	0.9935	0.9849	115.08	0.1087
PC (g GAE/L)	(3)	2.84 + 0.87∙A + 0.84∙t + 1.48∙B + 0.32∙A∙t + 0.64∙A∙B + 0.67∙t∙B − 0.35∙A^2^ − 0.50∙t^2^ − 0.38∙B^2^	6.67	0.9962	0.9906	177.11	0.3189
TPC (mg GAE/g EOP)	(4)	32.95 + 9.39∙A + 9.24∙t + 0.058∙B + 3.39∙A∙t − 7.61∙A∙B − 9.42∙t^2^	5.19	0.9922	0.9854	147.65	0.5122
HT (g/L)	(5)	0.48 + 0.13∙A + 0.13∙t + 0.22∙B + 0.049∙A∙t + 0.085∙A∙B + 0.10∙t∙B − 0.071∙A^2^ − 0.084∙t^2^ − 0.068∙B^2^	5.57	0.9969	0.9923	215.76	0.8571
HTC (mg/g EOP)	(6)	5.62 + 1.44∙A + 1.45∙t − 0.63∙B + 0.57∙A∙t − 0.94∙A^2^ − 0.88∙t^2^ − 0.37∙B^2^	5.98	0.9884	0.9769	85.44	0.7071
MAN (g/L)	(7)	3.09 + 0.84∙A + 0.89∙t + 1.81∙B + 0.35∙A∙t + 1.23∙A∙B + 0.74∙t∙B − 0.67∙t^2^	12.06	0.9776	0.9552	43.66	0.9776
MANC (mg/g EOP)	(8)	37.43 + 12.63∙A + 7.63∙t − 1.19∙B + 7.71∙A∙t + 2.66∙t∙B − 4.79∙A^2^ − 8.42∙t^2^	8.17	0.9764	0.9528	41.40	0.5030
FRAP (mg TE/g EOP)	(9)	42.34 + 15.06∙A + 8.50∙t − 3.01∙B − 2.97∙A∙B − 2.97∙t∙B − 8.99∙t^2^ − 8.12∙B^2^	5.53	0.9932	0.9852	124.69	0.5559
ABTS (mg TE/g EOP)	(10)	114.40 + 19.75∙A + 20.52∙t − 9.91∙B − 23.49∙A∙t − 18.03∙t^2^	11.17	0.8736	0.8104	13.83	0.6755

CV, coefficient of variation; FRAP, ferric reducing power; GAE, gallic acid equivalents; PC, phenolic concentration; HT, hydroxytyrosol concentration; HTC, hydroxytyrosol content; MAN, mannitol concentration; MANC, mannitol content; TE, Trolox equivalents; TPC, total phenolic content. A, amplitude (%); t, extraction time (min); B, solid loading (%, *w*/*v*). ^1^ *p*-value < 0.05.

**Table 4:** Predicted and experimental values obtained by ultrasound-assisted water extraction of exhausted olive pomace (EOP) under optimal conditions and comparison with milled EOP. Data represent the mean value ± standard deviation (*n* = 5).

Response Variable	Pelletized EOP ^1^			Milled EOP ^1^
	Predicted Values	Experimental Values	Error (%)	Experimental Values
Extraction Yield (%)	49.96	47.12 ± 0.45 ^a^	6.03	43.64 ± 0.04 ^b^
Phenolic concentration (g GAE/L)	5.07	4.60 ± 0.04 ^a^	10.21	4.42 ± 0.06 ^b^
Total phenolic compounds (mg GAE/g dry EOP)	43.04	40.04 ± 0.33 ^a^	7.50	38.44 ± 0.53 ^b^
Hydroxytyrosol concentration (g/L)	0.78	0.74 ± 0.03 ^a^	5.40	0.71 ± 0.01 ^a^
Hydroxytyrosol content (mg/g dry EOP)	6.77	6.42 ± 0.26 ^a^	5.45	6.19 ± 0.10 ^a^
Mannitol concentration (g/L)	6.13	5.86 ± 0.2 ^a^	4.60	5.44 ± 0.16 ^b^
Mannitol content (mg/g dry EOP)	52.39	50.92 ± 1.73 ^a^	2.88	47.34 ± 1.37 ^b^
FRAP (mg TE/g dry EOP)	53.25	50.95 ± 2.56 ^a^	4.50	49.92 ± 1.30 ^a^
ABTS (mg TE/g dry EOP)	105.80	100.64 ± 1.35 ^a^	5.13	95.59 ± 1.31 ^b^

FRAP, ferric reducing power; GAE, gallic acid equivalents; TE, Trolox equivalent. ^1^ The increment of temperature was 55 °C and 53 °C for pelletized EOP and milled EOP experiments, respectively. In each row, different letters (superscript) indicate significant differences between the data after applying a *t*-test (*p* < 0.05).

**Table 5:** Chemical and elemental composition (%, dry weight basis) of pelletized and milled exhausted olive pomace (EOP) after ultrasound-assisted water extraction under optimal conditions: 80% amplitude, 16 min, 11.5% (*w*/*v*) solid loading. Data represent the mean value ± standard deviation (*n* = 3).

Component	Raw (Pelletized) EOP ^1^	Extracted EOP Solid	
		Pelletized	Milled
Chemical characterization	%	%	%
Extractives	41.78 ± 1.85	20.98 ± 1.07	21.45 ± 0.26
Aqueous extractives	37.94 ± 1.89	14.89 ± 0.74	14.65 ± 0.40
Ethanol extractives	3.83 ± 0.16	6.09 ± 0.19	6.79 ± 0.66
Cellulose	9.67 ± 0.84	14.33 ± 0.64	15.71 ± 1.63
Hemicellulose	10.94 ± 0.53	15.16 ± 0.18	17.31 ± 1.51
Xylan	9.79 ± 0.53	14.70 ± 0.15	16.75 ± 1.48
Galactan	0.31 ± 0.31	1.24 ± 0.09	1.50 ± 0.31
Arabinan	1.82 ± 0.03	1.16 ± 0.03	1.27 ± 0.06
Mannan	0.42 ± 0.02	-	-
Acetyl groups	1.51 ± 0.17	1.30 ± 0.09	1.64 ± 0.19
Lignin	21.82 ± 0.89	32.32 ± 0.49	32.34 ± 0.73
Acid insoluble lignin	20.29 ± 0.68	31.54 ± 0.48	31.08 ± 0.72
Acid soluble lignin	1.54 ± 0.47	0.78 ± 0.07	1.26 ± 0.01
Ash	6.41 ± 0.21	1.62 ± 0.04	1.68 ± 0.12
Elemental analysis	%	%	%
Carbon	42.42 ± 0.24	49.52 ± 0.39	49.38 ± 0.37
Hydrogen	5.55 ± 0.08	6.21 ± 0.04	6.16 ± 0.04
Nitrogen	1.31 ± 0.06	1.57 ± 0.07	1.43 ± 0.07
Sulfur	ND	2.03 ± 0.23	2.27 ± 0.51

^1^ According to Gómez-Cruz et al. [28,57].

**Table 6:** Extraction yield and concentration of maslinic acid and oleanolic acid in the ethanolic extracts from each extracted solid of exhausted olive pomace obtained after the application of ultrasound-assisted water extraction in the Box–Behnken design.

Run	A	t	B	Extraction Yield (%)	Maslinic Acid (g/L)	Oleanolic Acid (g/L)
1	50	10	8.5	6.15 ± 0.06	0.77 ± 0.01	0.29 ± 0.00
2	50	10	8.5	5.65 ± 0.34	0.72 ± 0.03	0.26 ± 0.01
3	50	18	2	5.81 ± 0.63	0.70 ± 0.07	0.26 ± 0.03
4	50	18	15	5.49 ± 0.03	0.69 ± 0.01	0.26 ± 0.01
5	50	10	8.5	6.46 ± 0.15	0.81 ± 0.02	0.30 ± 0.01
6	80	10	15	5.80 ± 0.04	0.66 ± 0.01	0.24 ± 0.00
7	80	2	8.5	7.06 ± 0.23	0.64 ± 0.01	0.23 ± 0.01
8	50	2	2	6.13 ± 0.42	0.68 ± 0.05	0.25 ± 0.02
9	80	18	8.5	6.20 ± 0.34	0.81 ± 0.07	0.30 ± 0.02
10	50	2	15	8.18 ± 0.71	0.63 ± 0.03	0.23 ± 0.01
11	20	18	8.5	7.85 ± 0.73	0.79 ± 0.04	0.30 ± 0.01
12	50	10	8.5	8.62 ± 0.18	0.87 ± 0.01	0.33 ± 0.00
13	20	2	8.5	9.18 ± 0.66	0.62 ± 0.02	0.23 ± 0.01
14	50	10	8.5	8.67 ± 0.70	0.80 ± 0.07	0.30 ± 0.03
15	20	10	15	8.30 ± 0.04	0.67 ± 0.00	0.27 ± 0.00
16	80	10	2	5.64 ± 0.20	0.90 ± 0.02	0.33 ± 0.01
17	20	10	2	7.28 ± 0.18	0.72 ± 0.02	0.27 ± 0.00

A, Amplitude (%); t, extraction time (min); B, solid loading (%, *w*/*v*).

**Table 7:** Extraction yield and concentration of maslinic acid and oleanolic acid in the ethanolic extracts from raw exhausted olive pomace (EOP) and solids extracted from pelletized and milled EOP under optimal conditions of the ultrasound-assisted water extraction: 80% amplitude, 11.5% solid loading (*w*/*v*) and 16 min.

Components	Raw EOP	Extracted Solid EOP	
		Pelletized	Milled
Extraction Yield (%)	7.56 ± 0.78 ^a^	7.02 ± 0.18 ^a^	7.56 ± 0.12 ^a^
Maslinic acid concentration (g/L)	0.57 ± 0.00 ^b^	0.84 ± 0.04 ^a^	0.89 ± 0.01 ^a^
Oleanolic acid concentration (g/L)	0.21 ± 0.00 ^b^	0.32 ± 0.02 ^a^	0.34 ± 0.00 ^a^

In each row, different letters (superscript) indicate significant differences between the mean values after applying an analysis of variance (*p* < 0.05) and multiple comparison by LSD test.

## Data Availability

Data is contained within the article or supplementary material.

## Supplementary Materials

The following are available online at https://www.mdpi.com/article/10.3390/antiox10111781/s1, Figure S1: Response surface plot for the increment of temperature as function of the time and amplitude (solid loading was fixed at 8.5%, *w*/*v*) in the Box-Behnken design for the ultrasound-assisted water extraction of exhausted olive pomace, Figure S2: HPLC chromatogram (280 nm) of the aqueous extract obtained from pelletized (a) and milled (b) exhausted olive pomace after ultrasound-assisted water extraction at: 80% amplitude, 16 min and 11.5% solid loading (*w*/*v*), Table S1: F-ratio and *p*-value obtained for the operational parameters when the increment of temperature was evaluated as response variable in the Box–Behnken design for the ultrasound-assisted water extraction of exhausted olive pomace, Table S2: Statistical parameter values obtained for the extraction yield and concentration of maslinic acid and oleanolic acid after the ethanolic extraction (solid loading at 10%, *w*/*v*) of the extracted solids from exhausted olive pomace obtained after the application of ultrasound-assisted water extraction.
