# Supplementary material for: Sequential Extraction of Hydroxytyrosol, Mannitol and Triterpenic Acids Using a Green Optimized Procedure Based on Ultrasound
Source: Antioxidants (Basel). 2021 Nov 7;10(11):1781. doi: 10.3390/antiox10111781 (PMC8614775; doi:10.3390/antiox10111781)
Supplement: Supplementary file 1 [file antioxidants-10-01781-s001.zip › antioxidants-1420531-supplementary.pdf]

antioxidants

Article

Sequential Extraction of Hydroxytyrosol, Mannitol and Triterpenic Acids Using a Green Optimized Procedure Based on Ultrasound

Irene Gómez-Cruz[1,2], María del Mar Contreras[1,2*], Inmaculada Romero[1,2] and Eulogio Castro[1,2]

[1] Department of Chemical, Environmental and Materials Engineering, Universidad de Jaén, Campus Las Lagunillas, 23071 Jaén, Spain.
[2] Centre for Advanced Studies in Earth Sciences, Energy and Environment (CEACTEMA), Universidad de Jaén, Campus Las Lagunillas, 23071 Jaén, Spain.
* Correspondence: mcgamez@ujaen.es

Supplementary materials

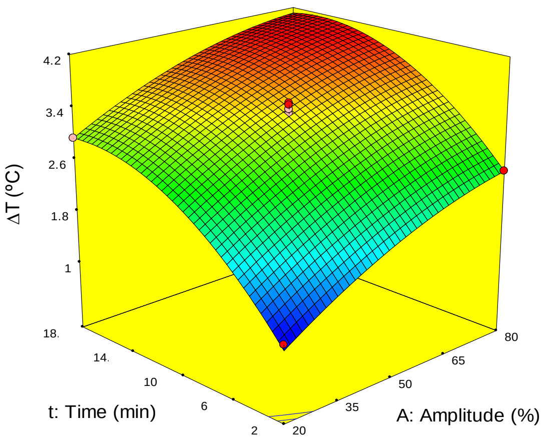

Figure S1. Response surface plot for the increment of temperature as function of the time and amplitude (solid loading was fixed at 8.5%, w/v) in the Box–Behnken design for the ultrasound-assisted water extraction of exhausted olive pomace.

Citation: Lastname, F.; Lastname, F.; Lastname, F. Title. *Antioxidants* **2021**, *10*, 1781. https://doi.org/10.3390/antiox10111781

Academic Editor: Firstname Lastname

Received: date
Accepted: date
Published: date

Publisher's Note: MDPI stays neutral with regard to jurisdictional claims in published maps and institutional affiliations.

Copyright: © 2021 by the authors. Submitted for possible open access publication under the terms and conditions of the Creative Commons Attribution (CC BY) license (https://creativecommons.org/licenses/by/4.0/).

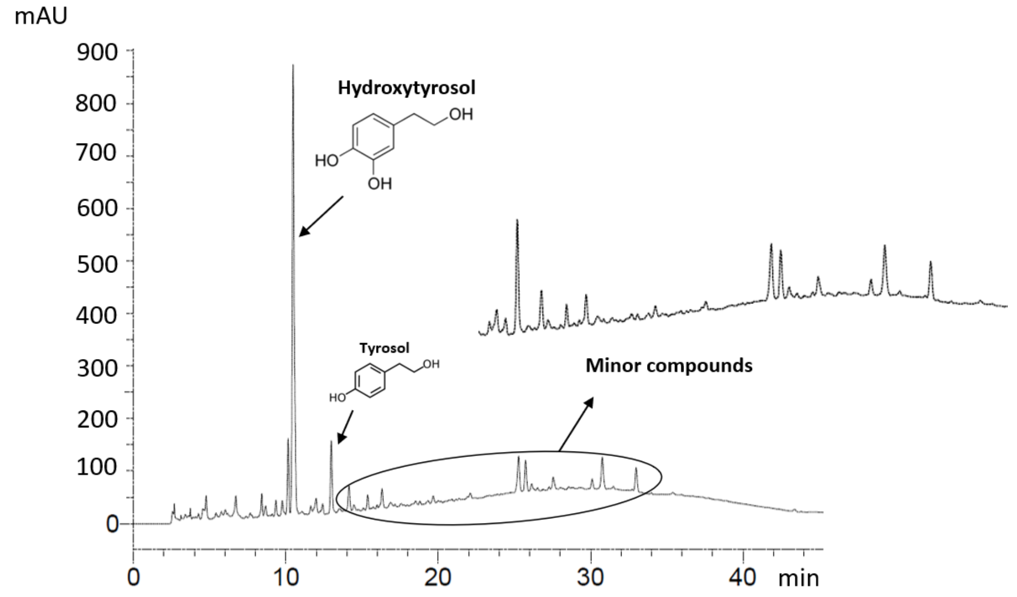

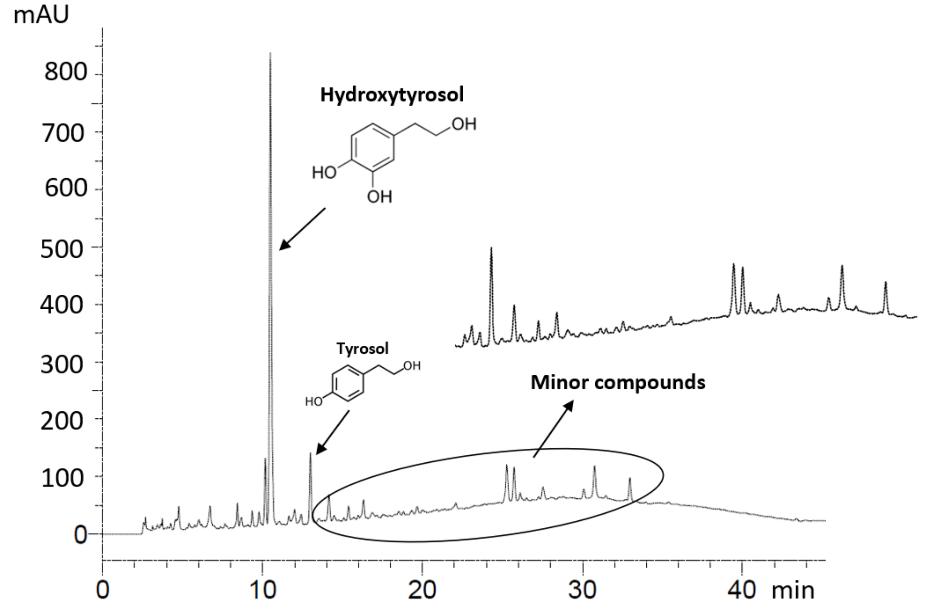

Figure S2. HPLC chromatogram (280 nm) of the aqueous extract obtained from pelletized (a) and milled (b) exhausted olive pomace after ultrasound-assisted water extraction at: 80% amplitude, 16 min and 11.5% solid loading (*w*/*v*).

Table S1. F-ratio and p-value obtained for the operational parameters when the increment of temperature was evaluated as response variable in the Box–Behnken design for the ultrasound-assisted water extraction of exhausted olive pomace.

Reponse variable	ΔT	
	**F-Ratio**	**P-Value**
A:Amplitude	470.80	< 0.0001
t:Time	862.70	< 0.0001
B:Solids	0.55	0.4838
AA	28.22	0.0011
At	3.17	0.1182
AB	0.78	0.4062

tt	178.98	< 0.0001
tB	0.39	0.5542
BB	1.87	0.2133

Table S2. Statistical parameter values obtained for the extraction yield and concentration of maslinic acid and oleanolic acid after the ethanolic extraction (solid loading at 10%, *w/v*) of the extracted solids from exhausted olive pomace obtained after the application of ultrasound-assisted water extraction.

	Yield (%)		Maslinic acid (g/L)		Oleanolic acid (g/L)	
**Variable**	**F-Ratio**	**P-Value**	**F-Ratio**	**P-Value**	**F-Ratio**	**P-Value**
A	6.60	0.0371	1.39	0.2777	0.42	0.5398
t	2.84	0.1356	5.19	0.0568	5.54	0.0508
B	0.89	0.3779	3.65	0.0978	2.19	0.1822
AA	0.59	0.4660	0.048	0.8323	0.015	0.9071
At	0.047	0.8348	0.0004	0.9847	0.0008	0.9784
AB	0.15	0.7064	2.24	0.1780	3.93	0.0878
tt	0.0096	0.9247	5.32	0.0544	6.28	0.0407
tB	1.19	0.3110	0.11	0.7497	0.25	0.6325
BB	2.05	0.1954	2.59	0.1518	1.99	0.2014

A, Amplitude (%); t, extraction time (min); B, solid loading (%, *w/v*).
